# Regulation of Bcl-2 Family Proteins in Estrogen Receptor-Positive Breast Cancer and Their Implications in Endocrine Therapy

**DOI:** 10.3390/cancers14020279

**Published:** 2022-01-07

**Authors:** Anna Kawiak, Anna Kostecka

**Affiliations:** 1 Intercollegiate Faculty of Biotechnology, University of Gdansk, Abrahama 58, 80-307 Gdansk, Poland; 2 Faculty of Pharmacy, Medical University of Gdansk, Hallera 107, 80-416 Gdansk, Poland; anna.kostecka@gumed.edu.pl

**Keywords:** apoptosis, Bcl-2, Bcl-xL, BH3 mimetics, estrogen receptor-positive breast cancer, estrogen receptor signaling, luminal breast cancer, Mcl-1

## Abstract

**Simple Summary:**

Breast cancer is the leading cause of cancer-related deaths in the female population, with estrogen receptor (ER)-positive breast cancer accounting for two-third of incidents. Endocrine therapy has proven beneficial in treating ER-positive breast cancer. However, resistance acquired toward therapy remains a drawback in treatment. The activation of signaling pathways regulated by estrogen receptors has been linked with the evasion of cell death and drug resistance. An important role in regulating signals determining cell survival or death is assigned to the Bcl-2 family of proteins. Since the upregulation of anti-survival Bcl-2 proteins has been associated with decreased endocrine therapy efficacy and resistance, this review focuses on the molecular regulation of this group of proteins in ER-positive breast cancer and their implications in endocrine therapy treatment. Furthermore, advancements in the development of agents targeting the Bcl-2 family proteins have been overviewed, and their application in ER-positive breast cancer is presented.

**Abstract:**

Estrogen receptor (ER)-positive breast cancer accounts for around two-thirds of breast cancer occurrences, with endocrine therapy serving as first-line therapy in most cases. Targeting estrogen signaling pathways, which play a central role in regulating ER+ breast cell proliferation and survival, has proven to improve patient outcomes. However, despite the undeniable advantages of endocrine therapy, a subset of breast cancer patients develop acquired or intrinsic resistance to ER-targeting agents, limiting their efficacy. The activation of downstream ER signaling pathways upregulates pro-survival mechanisms that have been shown to influence the response of cells to endocrine therapy. The Bcl-2 family proteins play a central role in cell death regulation and have been shown to contribute to endocrine therapy resistance, supporting the survival of breast cancer cells and enhancing cell death evasion. Due to the overexpression of anti-apoptotic Bcl-2 proteins in ER-positive breast cancer, the role of these proteins as potential targets in hormone-responsive breast cancer is growing in interest. In particular, recent advances in the development of BH3 mimetics have enabled their evaluation in preclinical studies with ER+ breast cancer models, and BH3 mimetics have entered early ER+ breast cancer clinical trials. This review summarizes the molecular mechanisms underlying the regulation of Bcl-2 family proteins in ER+ breast cancer. Furthermore, an overview of recent advances in research regarding the efficacy of BH3 mimetics in ER+ breast cancer has been provided.

## 1. Introduction

Breast cancer is the leading cause of cancer-related deaths in the female population [1]. Breast cancer is characterized by high heterogeneity reflected in the histological and molecular composition of the tumor and in treatment outcomes [2]. Immunohistochemical markers (IHC) are routinely used to guide the selection of treatment strategies and prognosis based on the presence of the estrogen receptor (ER), progesterone receptor (PR), human epidermal growth factor receptor 2 (HER2), and Ki67 proliferation index [3]. Advances in genomic analysis have identified diversity on the molecular level and introduced breast cancer intrinsic subtypes: luminal A, luminal B, HER2-enriched, and basal-like [4,5,6]. Based on gene expression patterns, luminal breast tumors have been divided into the luminal A and luminal B subtypes, which are distinguished by the higher expression of ER-related genes in the luminal A subtype and an increased proliferation signature in the luminal B subtype [7,8,9]. The overall and relapse-free survival rate among the breast cancer subtypes is the highest within the luminal A subtype [10]. Luminal B subtypes share similarities with the basal-like types regarding the proliferation marker index Ki67, which is the main distinguishing factor from the luminal A subtype [11]. A distinctive characteristic of the luminal B subtype is the expression of HER2/neu. Luminal B cancers account for 30% of HER2-overexpressing breast cancers, thus indicating the involvement of receptor tyrosine kinase-regulated signaling pathways such as PI3K/Akt and MAPK in cell proliferation [12,13].

Luminal breast cancers account for around two-thirds of breast cancers, and the estrogen receptor (ER) is a crucial marker guiding treatment. The strategies employed for the treatment of ER+ breast cancer target estrogen receptor signaling in the cell with the use of selective ER modulators (SERMS, e.g., tamoxifen), selective ER down regulators (SERDs, e.g., fulvestrant), or estrogen synthesis with aromatase inhibitors (AI, e.g., anastrozole) [14,15]. The effectiveness of anti-estrogen therapy has been demonstrated by responsiveness to treatment, longer relapse-free, and overall survival. However, 40–50% of patients do not respond to anti-estrogen therapy (de novo resistance) or develop resistance to treatment (acquired resistance) [16]. Research related to identifying novel mediators of the response to endocrine therapy has recognized downstream ER signaling mechanisms in therapy resistance. In particular, pro-survival mechanisms associated with the activation of ER signaling have been shown to decrease endocrine therapy efficacy.

A central role in regulating cell death is assigned to the Bcl-2 family of proteins, which orchestrate signals regulating the proliferation and survival of breast cancer cells. The pro-survival Bcl-2 proteins are overexpressed in ER+ breast cancer and are emerging as significant regulators of endocrine therapy resistance. In particular, Bcl-2 has been identified as an important prognostic marker across all breast cancer subtypes [17]. The expression of *BCL2* has been included in molecular panels determining the risk of recurrence and adjuvant treatment setting, such as Oncotype DX and PAM50-based–Prosigna Breast Cancer Prognostic Gene Signature Assay [18]. The tumorigenic potential of aberrant Bcl-2 protein expression was first associated with poor outcomes in non-Hodgkin’s lymphoma, [19] and Bcl-2 overexpression was further identified in various solid tumors. Interestingly, in breast cancer, the overexpression of Bcl-2 has been linked with the low-grade, slow-proliferating ER+ type and was associated with favorable outcomes [20]. In normal mammary epithelial cells, the expression of Bcl-2 is regulated by ER signaling, upregulated in malignant cells [21]. However, other events also contribute to Bcl-2 upregulation. *BCL2* amplification and copy number gains are uncommon in breast tumors, and there is no linear correlation between Bcl-2 gene transcripts and protein levels, thus pointing to post-transcriptional regulation of these proteins [17]. The mechanism of differential Bcl-2 protein expression in breast cancer is not fully elucidated, and understanding the mechanisms regulating Bcl-2 family protein expression in ER+ breast cancer could benefit treatment options. In this review, we provide an overview of the current knowledge regarding the Bcl-2 family of proteins in breast cancer regulation and the implications of targeting these proteins as a means of enhancing breast cancer endocrine treatment.

## 2. Estrogen Receptor Signaling

### 2.1. The Estrogen Receptor

**Figure 1:**
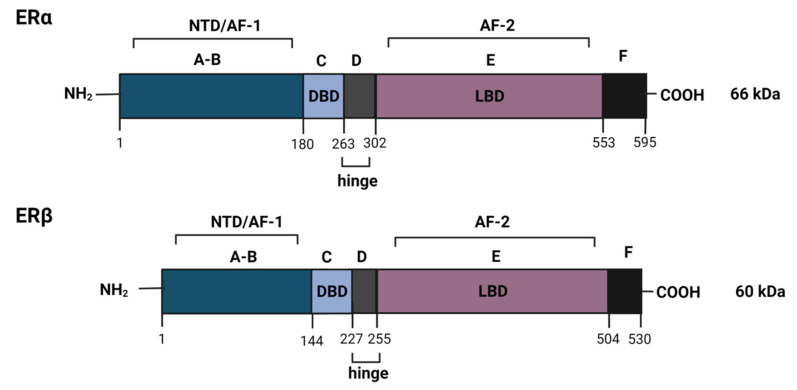
Structural organization of the α and β isoforms of the estrogen receptor (ER). The ER comprises six functional domains: A/B domains, N-terminal domain (NTD, containing activation function 1; C domain, the DNA-binding domain (DBD); D domain, hinge domain containing nuclear localization signals; E domain, ligand-binding domain (LBD) containing activation function 2; F domain, regulatory domain.

The estrogen receptor (ER) signaling pathways play a critical role in regulating the proliferation and survival of breast cells utilizing the female hormone estrogen in its activity. Estrogens are a group of steroid hormones that comprise estrone, estradiol, estriol, and estretrol. Estradiol (E2, 17β-estradiol) is most frequently referred to when referencing estrogen due to its predominance and significance in female reproduction. Estradiol is produced mainly by the ovaries, specifically the granulosa cells. Estriol (E3, 16-hydroxyestradiol) and estretrol (E4, 15α-hydroxyestriol) are produced during pregnancy by the placenta, whereas estrone (E1) is mainly produced during menopause [22]. Estrogens play a crucial role in growth, development, metabolism, and reproduction. Their activity is mediated through the association and activation of the estrogen receptors. Estrogen receptors belong to the nuclear receptor superfamily of transcription regulators and are present in two isoforms: α (ER α) and β (ER β) [23] (Figure 1). These receptors are encoded by genes located on different chromosomes, ERα encoded by the *ESR1* gene and ERβ by *ESR2*, containing evolutionarily conserved structural and functional domains [22]. The most conserved domain, the central DNA binding domain (DBD), shows 97% homology between ERα and ERβ. This domain recognizes estrogen response elements (ERE) located in promoters of target genes. The ligand-binding domain (LBD) is located at the C-terminal end and participates in receptor dimerization. The N-terminal domain is the most variable. Transcription mediated by ERα is carried out by two activation functions (AF), AF-1 located at the N-terminus, constitutively active, and AF-2 residing at the ligand-dependent C-terminal domain. The transcriptional activity of ERβ depends mainly on AF-2 and has reduced transcriptional activity due to the weaker function of AF-1 [24,25] (Figure 1). ERα and ERβ regulate the expression of different target genes upon the binding of ER ligands, estrogen, and anti-estrogens [26]. ERα and ERβ have been shown to induce opposing effects on the proliferation of ER+ breast cancer cells, with ERα promoting proliferation, and ERβ exhibiting antiproliferative activity [27]. Furthermore, ERβ represses the transcriptional activity of ERα [28] and modulates the activity of estrogen [29]. ERα and ERβ can also heterodimerize and alter ER-mediated gene expression [30].

### 2.2. Classical and Non-Classical Genomic ER Signaling Pathways

**Figure 2:**
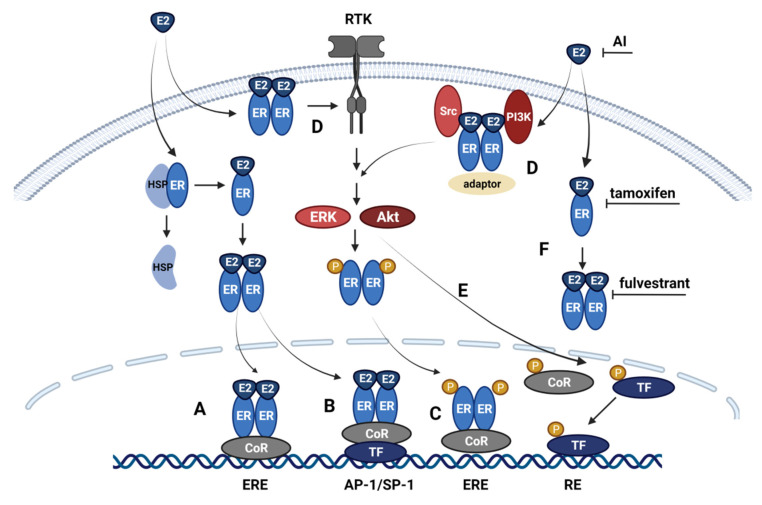
Mechanism of estrogen signaling and its inhibition by endocrine agents in breast cancer cells. (**A**) Classical genomic pathway. Estrogen (E2) upon binding to the estrogen receptor (ER), translocates to the nucleus, where it binds to estrogen response elements (EREs) within target genes. Coregulators (CoR) recruited to ER modulate gene expression. (**B**) Genomic, non-classical pathway. ER in the nucleus binds to target genes indirectly through interactions with transcription factors (TF). (**C**) Genomic, ligand-independent pathway. ER is activated by phosphorylation induced by kinases activated upon receptor tyrosine kinase (RTK)-mediated signaling. (**D**) Non-genomic, ligand pathway. Membrane-associated ER-bound with E2 activates RTKs and protein kinase cascades, which can also activate transcription factors modulating gene expression. (**E**) Convergence of non-genomic and genomic signaling. Activation of RTKs and protein kinases by ER-E2 complexes lead to the phosphorylation of ERs, coregulators, transcription factors regulating target gene transcription at multiple regulatory elements (RE). (**F**) Mechanism of endocrine agents. Aromatase inhibitors (AI) block estrogen production. Selective estrogen receptor modulators (SERMs), e.g., tamoxifen, competitively bind to ER, blocking the association with E2. Selective estrogen receptor modulators (SERDs), e.g., fulvestrant, inhibit ER dimerization. Created with BioRender.com (accessed on 5 December 2021).

ER signaling regulates gene expression and cellular processes through several pathways. The classical genomic pathway involves the binding of E2 to the ER, inducing conformational changes and releasing ERs from heat shock protein complexes. This leads to the dimerization of ER receptors, which translocate to the nucleus where they bind to estrogen response elements (ERE) within target genes [22,31]. Interactions with several coactivators and corepressors regulate ERα transcriptional output. Coactivators for ERα include, among others, members of the steroid receptor coactivator (SRC)/p160 group [32], whereas corepressors include the orphan nuclear receptor SHP (short heterodimer partner) and the tumor suppressor p53 [33] (Figure 2A). The transcription of genes by ER can also be mediated through the non-classical genomic pathway by its direct binding to DNA with transcription factors without binding to ERE. Transcription factors modulated by ER include AP-1, SP-1 or NF-κB [34,35] (Figure 2B). AP-1 (activator protein 1) is a transcription factor complex including Jun and Fos. The binding of ERα to AP-1 sites regulates the expression of genes involved in proliferation, such as cyclin D1 and IGF-I [29,36]. ER binding to AP-1 can be modulated through p-160/SRC coactivators [37]. Binding to the SP-1 site mediates the regulation of genes involved in proliferation and survival or apoptosis inhibition, such as cyclin D1 and Bcl-2 [34]. Furthermore, ER can repress the transcription of NF-κB by blocking the binding of NF-κB to DNA [35]. The activation of transcriptional activity of AP-1 and NF-κB is associated with endocrine therapy resistance [38,39].

### 2.3. Alternative ER Signaling Pathways

An alternative, genomic, non-ligand pathway regulated by ER is mediated by receptor tyrosine kinases (RTKs), epidermal growth factor receptor (EGFR), human epidermal growth factor receptor (HER2), and insulin-like growth factor receptor (IGF1-R) [40]. The activation of these receptors and downstream PI3K/Akt and MAPK/ERK signaling pathways induces the phosphorylation of ER. The Akt protein regulates ERα function through the activation of AF-1. This is achieved by phosphorylation on serines at positions 104, 106, 118, and 167 [41]. MAPK signaling similarly activates the AF-1 domain inducing the phosphorylation of serine 118 [42]. Furthermore, ERK and Akt can phosphorylate the coregulators of ER, further regulating ER-mediated transcriptional activity [43] (Figure 2C). Estrogens also exert their activity through non-genomic ER signaling, reported mainly for the activity of ERα, associated with rapid ER stimulation of signal transduction proteins and the activation of protein kinase cascades [16]. These non-genomic pathways include mobilization of intracellular calcium, stimulation of adenylate cyclase and cAMP signaling, activation of MAP kinase and PI3K/Akt signaling pathways. Activated ER can stimulate signaling pathways in the cell through direct interactions with protein kinases such as the Src kinase, the p85α regulatory subunit of PI3-kinase, Ras, G proteins, adaptor proteins Shc, scaffold proteins caveolin-1 [44]. The formation of activated ER complexes with Src and p85 PI3K subunit induces the MAPK and PI3K/Akt pathways [45]. Furthermore, membrane-associated ER can activate tyrosine kinase receptors. Interactions of ER with IGF-I, EGFR, and HER2/neu receptors trigger downstream MAPK and PI3K/Akt signaling [46,47,48,49] (Figure 2D). There are multiple mechanisms associated with non-genomic ER signaling. They depend on signaling pathways and their downstream targets in the cell, thus mediating diverse outcomes resulting from ER signaling [44]. Both the genomic and non-genomic pathways are complementary. Thus, both pathways can converge at multiple response elements regulating transcription mediated by ER. Non-genomic pathways can regulate the activity of transcription factors through phosphorylation mediated by protein kinases, therefore enabling ERs to regulate transcription at alternative diverse response elements [44] (Figure 2E).

The variety in ER-mediated signaling is additionally attributed to the heterodimerization of ERα and ERβ isoforms [30] as well as to the identified ERα and ERβ variants. Apart from the classical 66 kDa ERα (ERα66) and 60 kDa ERβ isoforms, several other protein variants have been recognized, which arise from alternative splicing or alternative promoters. The variant ERα46 (46 kDa isoform) regulates genomic estrogen signaling and competitively blocks ERα66 AF-1 activity through dimerization with this isoform [50]. The isoform ERα36 (36 kDa variant) localizes outside the nucleus and functions through the non-genomic pathway. ERα36 lacks both activation domains retaining only the DNA binding and ligand-binding domains. This variant also antagonizes the activity of ERα66 and has been associated with decreased sensitivity to tamoxifen [51]. Variants of the ERβ isoform have also been identified. However, their role in breast cancer is not fully elucidated [16]. The heterogeneity of the ER isoforms and their splice variants contribute to the complexity of ER signaling. The ERβ form possesses antiproliferative activity, and studies have suggested that the loss of ERβ expression contributes to breast tumor development [52,53]. Currently, only the ERα isoform is considered a clinical marker for patient treatment settings [54].

## 3. Mechanism of Anti-Estrogen Therapy

Since ER+ breast cancer is dependent on ERα for proliferation, therapy of this breast cancer subtype is directed toward disrupting ER signaling. Neoadjuvant or adjuvant endocrine therapy is the first-line systemic treatment for ER+ cancers and patients with nonmetastatic disease. Metastatic breast tumors are typically managed with endocrine therapy complemented with CDK4/6 inhibitors [3,55,56]. Therapy regimes include blocking the ligand–receptor interactions with ER antagonists or reducing estrogen production. Selective estrogen receptor modulators (SERMs), including tamoxifen and raloxifene, act as estrogen antagonists in breast cells [57]. Tamoxifen binds competitively to ER and blocks the binding of E2 to the receptor (Figure 2F). The activity of the AF-2 domain is inhibited, while the activation of the AF-1 is maintained. The complexing of tamoxifen and ER induces receptor dimerization and translocation to the nucleus, where it binds to the ERE site within promoters of ER responsive genes. The transcription of genes is inhibited partially due to the inactivity of AF-2. The binding of ER co-activators to AF-2 is also blocked. The activity of tamoxifen in breast cells is antagonistic due to the dependency of ER mainly on AF-2 in these cells [58]. In tissues like the uterus, tamoxifen acts as an agonist, where the dependency on AF-1 is higher [59]. Tamoxifen is metabolized by cytochrome P450 enzymes that convert tamoxifen initially to *N*-desmethyl-tamoxifen then to endoxifen. Alternatively, tamoxifen can be converted to the active metabolite 4-hydroxy-tamoxifen and further metabolized to endoxifen. 4-Hydroxy-tamoxifen and endoxifen have a higher potency than tamoxifen and are presumably responsible for the activity of tamoxifen in vivo [60]. The use of tamoxifen has been shown to reduce the risk of breast cancer by 38% in women and has been the therapy of choice in ER+ breast cancer, contributing to a significant increase in patient survival. However, around 40% of patients with adjuvant therapy develop resistance toward tamoxifen, and 50% of women with metastatic disease do not respond to treatment, and almost all metastatic patients relapse [16,60].

Another anti-estrogen antagonist, the selective estrogen receptor down-regulator (SERD), has been approved for ER+ cancer treatment and includes the ER antagonist fulvestrant (ICI 182,780). SERDs sequester ERα in the cytoplasm, inhibiting receptor dimerization, thus blocking genomic and non-genomic signaling of ERα (Figure 2F). SERDs block both AF-1 and AF-2 and completely inhibit the transcription of ER-responsive genes [61]. SERDs are anti-estrogens in all tissues and have a 100-fold greater affinity to ERα than tamoxifen [62]. They are used in metastatic breast cancer patients non-responsive to other anti-estrogen strategies [55]. In post-menopausal women, aromatase inhibitors (AI) like anastrozole, letrozole, exemestane are administered. AIs block the activity of aromatase and inhibit the production of estrogen in peripheral tissue and in tumor cells, thereby depriving the cell ligands for ER signaling [63] (Figure 2F). AIs predominantly inhibit the genomic rather than non-genomic pathway [55]. Intrinsic or acquired resistance can arise despite the treatment strategies used in ER+ cancers.

## 4. Bcl-2 Proteins in the Regulation of the Intrinsic Cell Death Pathway

**Figure 3:**
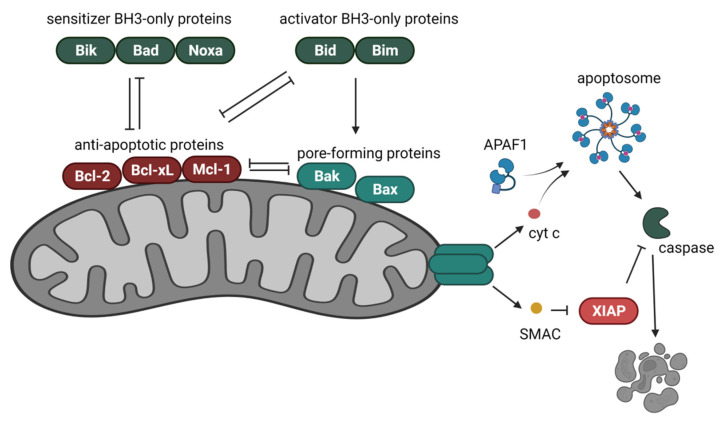
Bcl-2 family of proteins and their role in the intrinsic apoptotic pathway. The anti-apoptotic proteins (Bcl-2, Bcl-xL, Mcl-1) inhibit the activity of the pro-apoptotic proteins. The anti-apoptotic proteins are inhibited by sensitizer BH3-only proteins (Bik, Bad, Noxa) and activator BH3-only proteins (Bid, Bim). Activator BH3-only proteins, through binding to pore-forming proteins (Bax, Bak) induce their conformational change, oligomerization, and mitochondrial membrane permeabilization. This causes the release of cytochrome c, which associates with APAF1 to form the apoptosome activating procaspase-9. The apoptogenic factor SMAC released from the intermembrane space inhibits XIAP, an apoptosis inhibitor that binds caspases. Created with BioRender.com (accessed on 5 December 2021).

Apoptosis or programmed cell death is executed through two pathways: the extrinsic pathway mediated by death receptors and the intrinsic pathway referred to as the mitochondrial pathway. The intrinsic pathway is regulated by the Bcl-2 family of proteins, which control cell death induction mainly through regulating mitochondrial membrane permeability and the release of apoptogenic factors. The Bcl-2 family compromises the pro-apoptotic proteins, which increase membrane permeability and anti-apoptotic proteins, preventing membrane permeabilization. Based on the functions and structure of the Bcl-2 family of proteins, the group consists of the anti-apoptotic proteins (Bcl-2, Bcl-xL, Bcl-w, Mcl-1, A1), pore-forming pro-apoptotic proteins (Bax, Bak), the pro-apoptotic ‘BH3-only proteins’ (e.g., Bad, Beclin-1, Bid, Bik, Bim, Bmf, Noxa, Puma) [64]. The anti-apoptotic and pore-forming proteins contain four and three Bcl-2 homology (BH) domains, respectively, BH1 through BH4, whereas the ‘BH3-only proteins’ contain the BH3 domain. The BH3 domain is essential for the pro-apoptotic activity of proteins and the heterodimerization of pro-apoptotic and anti-apoptotic proteins [65]. BH3-only proteins, depending on their function, are divided into sensitizers and activators. The activator BH3-only proteins (Bid, Bim) induce the activity of the pore-forming Bax/Bak proteins through the binding of their BH3 domains. This leads to Bax/Bak conformational changes, their oligomerization, and subsequent pore formation. The sensitizer BH3-only proteins (Bad, Bik, Noxa) inhibit anti-apoptotic Bcl-2 family proteins through BH3 domain interactions. Sensitizer proteins do not activate Bak and Bax directly but inhibit anti-apoptotic Bcl-2 family proteins and render cells more prone to apoptotic stimuli. The interactions of sensitizer BH3-only proteins with anti-apoptotic proteins are competitive with activator BH3-only proteins and pore-forming proteins in terms of displacement from anti-apoptotic proteins [66] (Figure 3). Apart from the inter-regulation of Bcl-2 family proteins, they are also regulated through post-translational modifications, such as phosphorylation, dephosphorylation, proteolytic cleavage, and polyubiquitination [67].

The interactions of the Bcl-2 family proteins occur at the mitochondrial outer membrane (MOM), regulating its permeability. Activation of the effector Bax/Bak proteins determines the release of the apoptogenic factors from the intermembrane space. The activator BH3-only proteins recruit Bax proteins to the outer mitochondrial membrane. Bak is constitutively bound to the membrane and requires release from the anti-apoptotic proteins for activation [68]. Upon mitochondrial outer membrane permeabilization (MOMP), the proteins cytochrome c and SMAC are released from the intermembrane space. In the cytosol, cytochrome c binds monomers of the apoptotic protease-activating factor 1 (APAF1) and, in the presence of ATP or dATP, induces the oligomerization of APAF1 [69]. The APAF1 complex, upon a conformational change, reveals its caspase recruitment domain (CARD), forming the apoptosome that is capable of binding and activating procaspase-9. Once activated, caspase-9 cleaves and activates caspase-3 and caspase-7, resulting in the execution of apoptosis. Another apoptogenic factor released from the intermembrane space is SMAC (also referred to as DIABLO). SMAC neutralizes X-linked inhibitor of apoptosis protein (XIAP) and other IAPs. XIAP is an apoptosis inhibitor that binds caspase-3 and -7 [68] (Figure 3).

The Bcl-2 family proteins, due to their affinity to intracellular membranes, are located at various compartments within the cell. The most well-described localization, apart from mitochondria, is the endoplasmic reticulum. The Bcl-2 family proteins at the endoplasmic reticulum regulate calcium homeostasis and control cell death induced through the crosstalk between the endoplasmic reticulum and mitochondria [70].

## 5. Receptor Tyrosine Kinase (RTK)-Regulated Signaling in Anti-Estrogen-Induced Apoptosis

### 5.1. PI3K/Akt and MAPK Signaling

Pathways activated through receptor tyrosine kinase (RTK) signaling influence the activity of the Bcl-2 family of proteins. The overexpression and/or amplification of various types of RTKs, such EGFR, HER2, IGFR in breast cancer mediates signaling cascades that stimulate cell proliferation and cell survival. RTK signaling diverges into various signaling pathways, including the phosphatidylinositol 3-kinase/mammalian target of rapamycin (PI3K/mTOR) and the mitogen-activated protein kinase (MAPK) pathways [71]. The Ras oncoprotein, a small GTP-binding protein, plays a significant role in signal transduction from RTK receptors to downstream cellular targets promoting cell survival in breast cancer. Activated Ras induces the phosphorylation and activation of the intracellular kinase Raf, which further participates in signal transmission. Raf induces the activation of MEK1 and MEK2, which further phosphorylate the kinases ERK1 and ERK2. The ERK proteins activate a wide range of cellular targets, including cytoplasmic RSK (90 Kda ribosomal S6 kinase), which can further phosphorylate cellular substrates. Both ERK and RSK can translocate to the nucleus and activate transcription factors [72,73]. The PI3K/Akt/mTOR pathway also participates in breast cancer proliferation, and the survival and activation of this pathway mediate anti-estrogen resistance [55]. Signaling is transduced through PI3K, which is comprised of the p85 regulatory subunit and p110 catalytic subunit. The catalytic subunit is present in three isoforms p110α, p110β, p110δ. The p110α isoform is encoded by *PIK3CA*, the most frequently mutated gene in breast cancers. PI3K activates the serine/threonine kinase Akt, which is on the other hand repressed by PTEN, the loss of which is a common occurrence in breast cancer. The target of Akt is the kinase mTOR, which is present in two complexes mTORC1 and mTORC2 [55,74,75].

### 5.2. Regulation of Bcl-2 Family Proteins by RTK-Mediated Signaling

**Figure 4:**
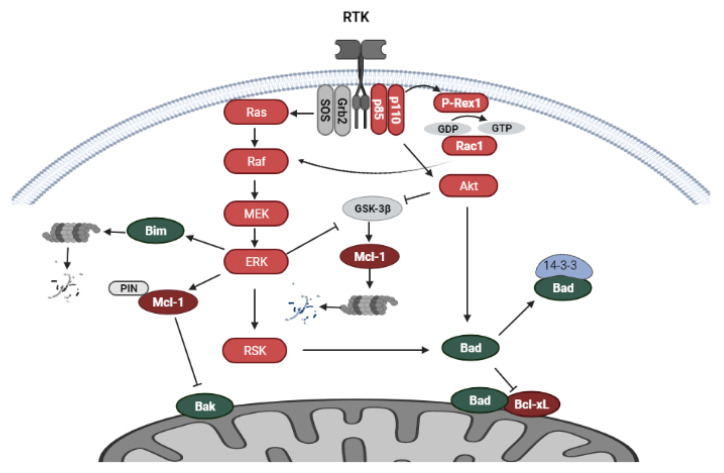
The regulation of Bcl-2 family proteins through PI3K/Akt and MAPK pathways in breast cancer cells. The BH3-only protein Bim is a target of ERK, which induces its phosphorylation leading to its proteasomal degradation. The activation of PI3K also leads to ERK-mediated Bim phosphorylation. This is facilitated through the guanine exchange factor for Rac1, P-Rex1, which induces Rac1/Raf/MEK/ERK/Bim signaling. Mcl-1 is a target of both ERK and Akt and is stabilized through the phosphorylation and inactivation of GSK3β. Additionally, ERK phosphorylates Mcl-1 enabling its stabilization by the isomerase Pin1 and inhibition of Bak. ERK and Akt also target the pro-apoptotic protein Bad. Phosphorylation of Bad recruits 14-3-3 proteins, which blocks its association with Bcl-xL at the mitochondria. Created with BioRender.com (accessed on 5 December 2021).

Non-genomic signaling of ER, through direct interactions with RTKs or pathway protein kinases, regulates Bcl-2 family protein activity and cell death induction. The Bcl-2 family proteins are under the regulation of the MAP kinase and PI3K/Akt/mTOR pathway. The ERK kinase modulates the activity of several Bcl-2 family proteins through phosphorylation of downstream targets. One such protein is the pro-apoptotic, BH3-only protein Bim, which sequesters Bcl-2 or its homologs. ERK inhibits the pro-apoptotic activity of Bim through its phosphorylation, which leads to proteasomal degradation resulting in cell survival [76]. Another target of ERK is Mcl-1, which is phosphorylated at two threonine residues, inducing its conformational changes and allowing the recognition of Mcl-1 by the isomerase Pin1, required for the stabilization of Mcl-1 [77]. Inhibition of ERK activity was shown to downregulate Mcl-1 levels and induce apoptosis in breast cancer cells [78]. One of the central kinases regulating the stability of Mcl-1 is GSK-3β, which through the phosphorylation of Mcl-1, targets it for proteasomal degradation [79]. The PI3K/Akt and MAPK pathways have been shown to increase the stability of Mcl-1 through the phosphorylation and inactivation of GSK-3β in breast cancer cells [80]. Furthermore, ERK and Akt upregulate the transcription of Mcl-1 [81]. A pro-apoptotic protein regulated by both ERK and Akt is the BH3-only protein Bad. The phosphorylation of Bad recruits 14-3-3 proteins, thus blocking the pro-apoptotic activity of Bad at the mitochondrial membrane through dissociation from Bcl-xL (Figure 4) [82]. The prolonged activation of PI3K/Akt signaling induces the phosphorylation of Bad at Ser 136, whereas prolonged activation of MAPK/ERK mediates phosphorylation at Ser 112 [83].

ER signaling has also been shown to mediate the crosstalk between the PI3K/Akt and MAPK/ERK pathways in the regulation of the Bcl-2 family proteins. Estrogen inhibited apoptosis induction in MCF-7 breast cancer cells through upregulation of PI3K/Akt and MAPK/ERK signaling and phosphorylation of Bad at the Akt and ERK phosphorylation sites (Ser 136 and Ser 112). Interestingly, the inhibition of PI3K abrogated estrogen-induced ERK activation and phosphorylation of Bad at Ser 112, the ERK phosphorylation site, indicating crosstalk between PI3K/Akt and MAPK/ERK signaling upon estrogen stimulation. Furthermore, Ras was found to be the target of estrogen-activated PI3K/Akt and MAPK signaling, as dominant negative Ras blocked estrogen-induced Bad phosphorylation [83]. Another study indicated that in certain cell lines with *PIK3CA* gain-of-function mutations and/or *HER2* overexpression, the inhibition of PI3K upregulated Bim through MAPK signaling inhibition not involving Ras. The crosstalk between PI3K and MAPK signaling was identified through P-Rex1–dependent activation of Rac1. P-Rex1, a PI(3,4,5)P3-dependent guanine exchange factor for Rac1, induces Rac1/Raf/MEK/ERK/Bim signaling (Figure 4). Importantly, PI3K/Akt and Rac/ERK pathway inhibition were required for efficient apoptosis induction by the PI3K inhibitor [84].

### 5.3. Endocrine Therapy and RTK-Mediated Signaling

Suppression of RTK-mediated downstream signaling has been shown to enhance anti-estrogen therapy. The PI3K/Akt signaling pathway, in particular, is an important target in ER+ breast cancer as PI3K deregulation through mutation of the catalytic subunit alfa of PI3K (*PIK3CA*) is a common occurrence in breast cancer and correlates with the ER+ status [85]. In long-term estrogen deprived (LTED) breast cancer cells, the upregulation of PI3K/Akt induced the levels of, among others, Bcl-xL and p-Bad (Ser136), enabling cell survival [86]. In LTED breast cancer xenografts, PI3K/Akt upregulation stimulated tumor growth resulting in a more aggressive and hormone-resistant phenotype. The crosstalk between the ER and PI3K pathways allows cells to adapt to estrogen deprivation or grow in the presence of anti-estrogens through the upregulation of ER signaling and protein signaling kinases sustaining transcription and cell survival [87]. Bidirectional crosstalk between ER and RTK-mediated signaling has been suggested as an important factor contributing to endocrine resistance. Increased RTK activation induces ER signaling, which through genomic and non-genomic signaling, in turn, reactivates RTK-activated pathways. This crosstalk reinforces signaling through both ER and RTK-mediated signaling, suggesting the dual inhibition of these pathways for increased inhibition of breast cancer cell proliferation [40]. Accordingly, the use of a PI3K inhibitor (wortmannin) in combination with anti-estrogens, the selective estrogen receptor modulator tamoxifen, or the selective estrogen receptor down-regulator fulvestrant, resulted in increased tumor suppression to a greater extent than either inhibitor alone [87]. Further research supported these findings and indicated that PI3K/mTOR inhibition induced breast cancer cell apoptosis, thereby preventing the emergence of hormone-independent cells. The research suggested that early intervention with combination therapy targeting ER and PI3K signaling could abrogate acquired resistance to anti-estrogens in ER+ tumors with increased PI3K signaling (harboring *PIK3CA*, *PTEN*, or *HER2* mutations) [88].

Apart from PI3K/Akt activation, breast cancer cell adaptation to long-term estrogen deprivation upregulates the MAPK pathway [89,90]. Long-term tamoxifen exposure facilitates the translocation of ERα from the nucleus to the cytoplasm and activates EGFR-induced MAPK signaling. Interactions of ERα with the kinase c-Src were found to enable the activation of EGFR by ERα. Blockade of c-Src activity restored the sensitivity of cells to tamoxifen, as did blockade of the EGF receptor and downstream MAPK signaling [91]. Long-term AI treated (letrozole) breast cancer xenografts showed an upregulation of HER2, increased ERα expression, and MAPK signaling. The treatment with letrozole initially reduced tumor growth through suppressing estrogen synthesis but upregulated ERα and activated HER2 and MAPK signaling. Long-term treatment eventually resulted in acquired resistance to letrozole, and the expression of ERα was decreased in proliferating cells. However, the expression of the adaptor protein Grb2 was upregulated. Grb2 links activated RTKs to a guanine nucleotide exchange factor (SOS) mediating signal transduction to Ras, thus further inducing MAPK activity. The use of a MAPK inhibitor or growth factor receptor inhibitor, gefitinib, restored the sensitivity of tumors to endocrine agents. Furthermore, the combination of gefitinib with letrozole yielded better results than either drug alone, indicating the benefits of blocking both ER and growth factor-mediated signaling in ER+ breast cancers [92].

The ability of an inhibitor to efficiently induce apoptosis is a strong predictor for antitumor activity in vivo [93]. The inhibition of PI3K/Akt and ERK signaling in breast cancer cells upregulated Bim. However, apoptosis was induced in a fraction of the population [84]. In another study, the inhibition of PI3K induced the levels of the pro-apoptotic Bim in *PIK3CA*-mutant cells. However, apoptosis was not induced. It was suggested that a population of available Bcl-2 proteins could sequester Bim, blocking its pro-apoptotic activity. The addition of a BH3 mimetic ABT-263, which targets Bcl-2, Bcl-xL, and Bcl-w, significantly enhanced apoptosis induction in these cells, indicating the beneficial role of targeting Bcl-2 proteins alongside PI3K inhibition [94].

## 6. Endoplasmic Reticulum in the Regulation of Anti-Estrogen Induced Apoptosis

### 6.1. Regulation of Bcl-2 Family Proteins at the Endoplasmic Reticulum

**Figure 5:**
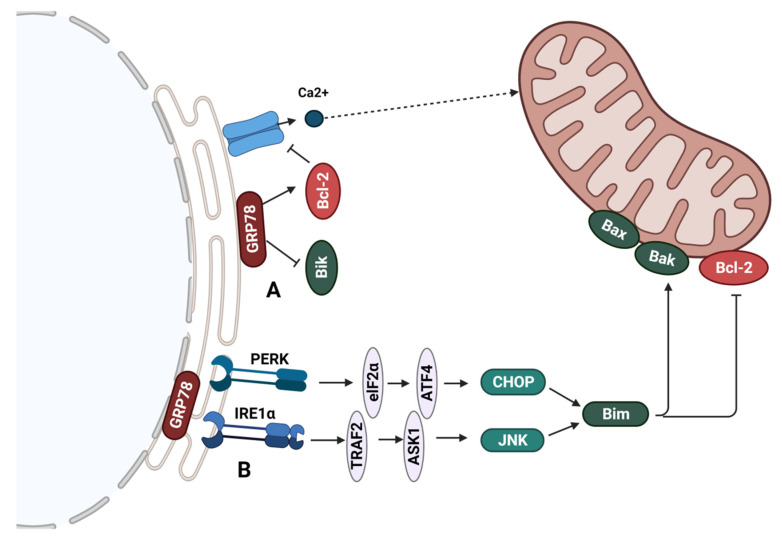
Regulation of apoptosis induction by Bcl-2 family proteins at the endoplasmic reticulum in endocrine-resistant breast cancer cells. (**A**) In the presence of high GRP78/BiP levels, Bik is sequestered by GRP78. Downregulation of GRP78 releases Bik, which binds Bcl-2 and blocks its antiapoptotic activity, enabling the release of Ca^2+^ from the ER and apoptosis induction at the mitochondria (**B**). In the presence of low levels of GRP78, induction of the UPR and activation of IRE1α and PERK leads to cell death. IRE1α activates JNK via the activation of TRAF2 and ASK1. JNK induces apoptosis through the upregulation of Bim, which can further activate Bax/Bak. PERK phosphorylates and inactivates elF2α resulting in the induction of ATF4 and CHOP, which has multiple downstream targets such as Bim. Created with BioRender.com (accessed on 5 December 2021).

The endoplasmic reticulum also regulates cell death through the activity of Bcl-2 proteins residing at the ER. The BH3-only protein Bik, located primarily at the endoplasmic reticulum, is implicated in anti-estrogen-induced apoptosis [95]. Bik induces apoptosis through sequestering Bcl-2, blocking its anti-apoptotic activity and enabling the release of Ca^2+^ from the ER. Bik-mediated Ca^2+^ release activates apoptosis at the mitochondria. The activity of Bik is regulated at the endoplasmic reticulum by the glucose-regulated protein GRP78 [96]. GRP78 (also referred to as BiP) is a major chaperone at the endoplasmic reticulum and a member of the HSP70 family of chaperones. GRP78 as an endoplasmic reticulum stress regulator is involved in the unfolded protein response (UPR), controlling proper protein folding and directing unfolded proteins for degradation. GRP78 regulates Ca^2+^ binding and participates in the activation of endoplasmic reticulum stress inducers [97]. The levels of GRP78 have been shown to be upregulated in many cancers, including breast cancer [98]. Increased GRP78 levels in cancer cells have been associated with changes in tumor cell metabolism, such as elevated glucose consumption and impaired protein glycosylation. High glucose and oxygen utilization facilitate the accumulation of misfolded proteins activating UPR for cell survival [99]. The anti-apoptotic activity of GRP78 has been associated with its ability to bind Bik. GRP78, and not other endoplasmic reticulum chaperones, has been shown to selectively complex with Bik [96]. The complex formation with Bik inhibits its pro-apoptotic activity, presumably through altering its conformation or interference with the heterodimerization with anti-apoptotic proteins. The sequestering of Bcl-2 by Bik leads to Bax activation and cytochrome c release from the mitochondria [100] (Figure 5A). Our results and those of others showed that silencing GRP78 expression upregulates Bik levels in breast cancer cells [96,101].

### 6.2. GRP78-Mediated Sensitivity of ER+ Breast Cancer to Endocrine Therapy

The inhibition of GRP78 activity was shown to sensitize ER+ breast cancer cells to tamoxifen-mediated cell death induction [101]. GRP78 has been implicated in anti-estrogen resistance. The levels of GRP78 are frequently upregulated in refractory tumors, and activation of the UPR pro-survival mechanism has been reported to be a key factor contributing to the development of anti-estrogen mediated resistance in breast cancer. Cell lines resistant to endocrine therapy have displayed increased GRP78 levels, and this was confirmed in anti-estrogen resistant breast cancer xenografts. Long-term estrogen withdrawal leads to GRP78 activation and its sequestering of Bik. GRP78 downregulation sensitizes anti-estrogen resistant tumors and cell lines to anti-estrogens such as tamoxifen and fulvestrant [102,103]. The involvement of GRP78 in anti-estrogen resistance has been associated with acquired resistance to anti-estrogens, not de novo resistance [102]. Since two-thirds of breast cancer patients demonstrate GRP78 upregulation, research suggests that in this subset of breast cancers, the increased expression of GRP78 will block the pro-apoptotic functions of Bik, thus inhibiting cell death mediated by anti-estrogen deprivation [96].

Studies have implicated the connection between reticulum stress and the therapeutic activity of estrogen. Despite the tumor-promoting effects of estrogen in ER+ breast cancer, studies have demonstrated that long-term estrogen deprivation or anti-estrogen treatment can resensitize cells to cell death-inducing effects of estrogen. In LTED cells, estrogen was found to reactivate ER signaling leading to cell death induction. The mechanism of ER-induced cell death was linked with the upregulation of the UPR response [104]. Although UPR activation is a pro-survival mechanism, prolonged activation of UPR can result in cell death. Upon UPR induction, GRP78 is released from three signaling control UPR components: IRE1α, ATF6, and PERK in order to assist in protein folding. The release of these unfolded protein sensors enables downstream activation of UPR. Prolonged stress leads to pro-death mechanisms that involve the induction of DNA-damage inducible transcript 3 (CHOP) and IRE1α mediated activation of c-Jun terminal kinase (JNK) [105]. The recruitment of TNF receptor-associated factor 2 (TRAF2) to IER1α triggers apoptotic pathway induction through activation of ASK1 and JNK. JNK participates in apoptosis induction by phosphorylating Bcl-2 family proteins modulating their activity [106]. Targets of JNK include BH3-only proteins such as Bim, activated upon phosphorylation, and Bcl-2, which loses its anti-apoptotic activity [107]. CHOP is activated by all three arms of the UPR as it possesses binding sites for ATF6, ATF4 (a downstream target of PERK signaling), and XBP1 (a downstream target of IRE1α) [106]. CHOP promotes apoptosis through the downregulation of Bcl-2 [105] and upregulation of Bim [108]. Estrogen was found to upregulate the stress sensors IRE1α and PERK in LTED cells, preceding the increase in pro-apoptotic proteins CHOP and Bcl-2 family proteins such as Bim [104] (Figure 5B). Since GRP78 has been found to desensitize IRE1α to low-stress levels [109], decreased levels of GRP78 may contribute to increased activity of IRE1α and, in consequence, cell death. Accordingly, estrogen-mediated UPR and cell death induction in LTED cells was associated with low basal proteasomal activity and GRP78 levels. LTED MCF-7 cells expressed lower levels of GRP78 than the parental MCF-7 cells, and a weaker induction in these levels upon estrogen was observed. The increased sensitivity of LTED cells and tumors to estrogen-induced apoptosis was mainly associated with increased ER signaling, thus linked to tumors with genomic amplification of *ESR1*. A case study showed partial regression of tumor growth in a metastatic breast cancer patient with *ESR1* amplification upon estrogen therapy [110]. These cell death-promoting effects of estrogen treatment were supported by a study showing tumor regression in patient-derived xenografts from hormone-resistant metastatic breast cancer [111]. Thus research suggests that anti-estrogen resistant breast tumors with *ESR1* amplification, identified in almost 20% of metastatic ER+ breast cancers [112], as well as decreased levels of protein-folding chaperones, may be targeted by estrogen (17β-estradiol) treatment [104].

## 7. Bcl-2 Family Proteins in Breast Cancer

### 7.1. Bcl-2

Overexpression of Bcl-2 has been observed in various breast cancer subtypes. In a prospective analysis including over 110,000 early-stage breast cancer cases, Bcl-2 upregulation was identified in 73% of breast cancers, with 86% determined in the ER+ subtype [17]. In line with these findings in a gene expression dataset analysis comprising around 2000 breast cancers, Bcl-2 was most distinctly upregulated in luminal breast cancers [113,114]. Bcl-2 has been shown to be an important prognostic marker in breast cancer patients and is associated with a favorable outcome [115]. In a meta-analysis comprising 17 breast cancer studies, Bcl-2 was associated with improved disease-free survival (DFS) and overall survival (OS). This prognosis was independent of lymph node status, tumor size, and tumor grade, as well as a range of other biological variables [116]. This favorable prognostic of Bcl-2 could be attributed to the regulation of *BCL-2* transcription by estrogen. *BCL2* is an estrogen-responsive gene, and estrogen induces *BCL2* expression via two estrogen-responsive elements located within its coding region leading to apoptosis inhibition [117]. Thus Bcl-2 expression could be indicative of increased estrogen signaling in luminal breast cancers, which upon anti-estrogen treatment would be reduced along with cancer cell proliferation [18]. The favorable prognostic value of Bcl-2 could also be attributed to the higher Bcl-2 load on the mitochondria and mitochondrial priming, described in the next chapter. The positive prognostic value of Bcl-2 has been mainly associated with early-stage breast cancer rather than advanced/metastatic cancer resistant to therapy [18].

### 7.2. Bcl-xL

In contrast to Bcl-2, Bcl-xL is associated with higher tumor grade and increased number of positive nodes and is a predictor of worse overall survival [118]. The Bcl-xL protein is associated mainly with invasive breast cancer and has been shown to be overexpressed in 43% of invasive breast tumors [118]. In mouse mammary epithelial cells, Bcl-xL overexpression did not influence tumor formation; however, it did promote metastasis [119]. The pro-metastatic activity of Bcl-xL was associated with its ability to induce resistance to TGF*β*1-induced apoptosis. This induced anchorage-independent growth and increased cell survival in the circulation of mice with Bcl-xL overexpressing breast tumors [120]. Further studies indicated that the metastatic function of Bcl-xL was independent of its anti-apoptotic activity but relied on the nuclear activity of Bcl-xL and its ability to induce epigenetic modifications of the TGFβ promoter and increased TGFβ signaling [121]. Another study revealed that cell migration promoted by Bcl-xL was related to mitochondria-associated Bcl-xL and its influence on VDAC1 permeability at the mitochondrial membrane that promoted ROS production by the electron transport chain [122].

### 7.3. Mcl-1

Other Bcl-2 family proteins co-expressed alongside Bcl-2 in ER+ breast cancer include Mcl-1. Mcl-1 protein expression has been found to be elevated in breast cancers, with the highest levels determined in estrogen receptor-positive breast tumors [123]. High levels of Mcl-1 have been associated with high tumor grade and poor prognosis in breast cancer patients [80]. Studies have confirmed the functional role of Mcl-1 in breast tumor development and revealed Mcl-1 expression is a necessity in breast tumorigenesis [124]. Mcl-1 expression at different stages of mammary tumorigenesis in the *MMTV-PyMT* mouse model resembling the progression and morphology of human breast cancers showed that tumors of mice with homozygous deletion of Mcl-1 in the mammary epithelium expressed equally high levels of Mcl-1 as those in WT tumors. This indicated a selective pressure against *MCL1* loss in mammary tumors. Silencing *MCL1* in breast tumor xenografts reduced tumor growth. However, in end-stage tumors, Mcl-1 expression had recovered, showing its requirement for tumor development [124]. In breast cancer cell lines, Mcl-1 expression was found necessary for the survival of 47% of breast cancer cells irrespective of the subtype [125]. Furthermore, *MCL1* expression and amplification exceeded that of *BCL2* and *BCL2L1* (Bcl-xL) in clinical ER+ breast cancer samples [123]. ERα signaling has been associated with the regulation of Mcl-1 expression. In an E2-dependent manner, ERα upregulates Mcl-1 through binding to a half ERE site within the Mcl-1 promoter in a complex with SP-1 transcription factor [126].

## 8. Targeting Bcl-2 in Breast Cancer with BH3 Mimetics

### 8.1. BH3 Mimetics

The frequent upregulation of pro-survival Bcl-2 proteins in luminal breast cancers suggests that these cancers could benefit from inhibitors targeting their activity. The most promising strategy in the targeting of Bcl-2 proteins has been the development of BH3 mimetics. BH3 mimetics disrupt complexes between BH3-only proteins and anti-apoptotic Bcl-2 proteins through the binding to the hydrophobic groove of anti-apoptotic proteins mimicking the activity of BH3-only proteins [127]. The binding of BH3 mimetics to anti-apoptotic leads to the release of BH3-only proteins, which are then able to activate Bax/Bak. The requirements for a BH3 mimetic are the binding to at least one Bcl-2 family protein at a nanomolar concentration and subsequent cell death induction in a Bak or Bax-dependent manner [128]. One of the first identified BH3 mimetics was the natural compound gossypol and its derivative AT-101, which shows affinity to and binds Bcl-2, Bcl-xl, and Mcl-1. These compounds, however, displayed pleiotropic activity [129]. Efforts are at present directed to develop ‘on target’ BH3 mimetics. BH3 mimetics evaluated toward breast cancer cells, include the compounds ABT-737, ABT-263 (navitoclax) and ABT-199 (venetoclax) [18]. ABT-737 and ABT-263 bind with high affinity to Bcl-2, Bcl-xL, and Bcl-w but display no affinity towards Mcl-1. These mimetics induce the release of Bax and Bak from Bcl-2 and Bim from Bcl-2 [130]. ABT-199 selectively binds to Bcl-2 and not to Bcl-xL or Bcl-w. The use of BH3 mimetics in ER+ breast cancer cells was found to increase the efficacy of various therapeutic agents through targeting pro-survival Bcl-2 proteins (Table 1). ABT-263 synergistically increased the antiproliferative effects of various chemotherapeutic agents in MCF-7 cells [131]. In a preclinical study with patient-derived xenograft models of ER+ primary breast cancer, BH3 mimetics ABT-737 and ABT-199 enhanced the responsiveness of tumors to tamoxifen. Three PDX models, which corresponded to the luminal B subtype and expressed high levels of Bcl-2 and Bcl-xL, were analyzed. Complete tumor regression was observed in one PDX model upon combination treatment. In the other two PDX models that exhibited a partial response to combination therapy, an increase in p-Akt was observed. However, these tumors did not harbor *PIK3CA*, *AKT1*, or *PTEN* mutations but *TP53* mutations. A PI3K/mTOR inhibitor combined with ABT-737 and tamoxifen markedly attenuated tumor growth in these tumors [114]. The benefits of employing a PI3K/mTOR inhibitor in *TP53* mutants were shown in a recent study, where a decrease in the proliferation of *TP53* mutant, triple negative breast cancer cells was achieved through mutp53 degradation by PI3K/mTOR inhibition [132]. In ER+ breast cancer cells harboring *PIK3CA* and *PTEN* mutations, treatment with a PI3K/mTOR inhibitor resulted in increased RTK-mediated signaling and upregulation of EGFR/ERK/p-Bad^S112^. The use of ABT-737 enhanced cell death in combination with the PI3K/mTOR inhibitor [133].

### 8.2. Mitochondrial Priming for BH3 Mimetic Activity

**Figure 6:**
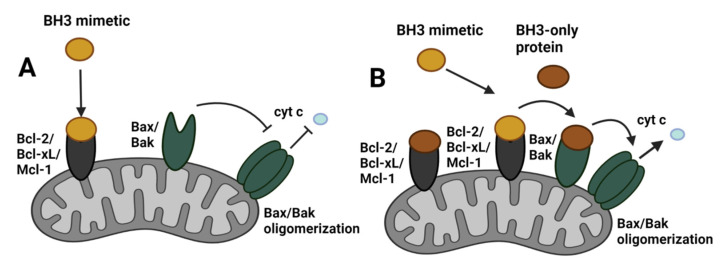
Priming of mitochondria for the activity of BH3 mimetics. (**A**) In the unprimed state, the low BH3-only protein load does not lead to apoptosis induction upon BH3 mimetic binding (**B**). In the primed state, high BH3-only protein load enables translocation of the BH3-only protein upon the binding of the BH3 mimetic, which leads to Bax/Bak activation, oligomerization, and cytochrome c release. Created with BioRender.com (accessed on 5 December 2021).

BH3 mimetics exert minimal effects in ER+ breast cancers as single agents. Mitochondrial priming through Bcl-2 family protein upregulation and complex formation is a prerequisite for their activity. Increased Bcl-2 levels frequently lead to induced equivalent levels of their partner BH3-only proteins [18]. The complexing of BH3-only proteins with their anti-apoptotic binding partners protects them from proteasomal degradation [142]. The high levels of pro-apoptotic proteins render the cell sensitive to BH3 mimetics, which disrupts the binding between the anti-apoptotic and corresponding BH3-only protein. The release of the BH3-only protein enables the activation of the Bax/Bak proteins [143] (Figure 6). Anti-estrogens and other agents used in ER+ breast cancer treatment such as PI3K/mTOR inhibitors and chemotherapeutics have been reported to upregulate Bcl-2 proteins, priming the cell for BH3 mimetic activity [114,130,144]. Tamoxifen upregulated Bcl-2 in PDX models of ER+ breast cancer, whereas ABT-737 and ABT-199 were found to elicit a response in PDX xenografts only in combination with tamoxifen, suggesting the requirement of Bcl-2 upregulation with tamoxifen for the activity of these BH3 mimetics [114]. PI3K/mTOR inhibitors were shown to upregulate Bcl-2 levels in MCF-7 breast cancer cells and synergize with the BH3 mimetics ABT-737 and ABT-199 [114]. Similarly, T47D cells displayed an adaptive response to the PI3K/mTOR inhibitor BEZ235 with Bcl-2 and Bcl-xL upregulation and were sensitized with ABT-737 treatment [133]. In paclitaxel-resistant MCF-7 cells, Bcl-2, and Bcl-xL levels were upregulated, and ABT-737 restored the sensitivity of resistant cells to paclitaxel [134]. The sensitivity of cells to Bcl-2 inhibition has been shown to be associated with the complexing of Bcl-2 with Bim. Bcl-2 primed with Bim (activator BH3-only protein) enables the pro-apoptotic activity of BH3 mimetics such as ABT-737 and ABT-199. The binding of the BH3 mimetic to Bcl-2 releases Bim, which interacts with Bak or Bax [145,146]. In PDX models of ER+ breast cancer, tamoxifen was found to increase the levels of Bcl-2 and Bim, whereas ABT-737 disrupted these complexes [114]. Bim/Bcl-2 complexes were also found to be a prerequisite in the chemosensitization of luminal breast cancer cells to taxanes by ABT-737. In this study, using a PDX model of primary breast cancer combination therapy was associated with the release of Bim from Bcl-2 complexes and induction of cell death. This indicated that the presence of Bim in association with Bcl-2 primed cells for cell death induced by ABT-737 [130].

### 8.3. Venetoclax and ER+ Breast Cancer

Navitoclax has displayed promising anti-tumor effects and has entered clinical trials against lymphomas. ABT-263 (navitoclax) displays similar potency to ABT-737 but is orally bioavailable due to its better physicochemical and pharmaceutical properties. Navitoclax, however, has been reported to induce dose-limiting thrombocytopenia, associated with the dependence of platelets on Bcl-xL for survival [147]. In order to avoid these side-effects of Navitolax, the BH3 mimetic ABT-199 (venetoclax) was designed. Venetoclax selectively binds with high affinity to Bcl-2, but not to Bcl-xL or Bcl-w, thus does not affect platelets. Venetoclax has been shown to be effective in xenograft models of human lymphoid tumors that overexpress Bcl-2, a crucial protein for the survival of these cancers. The selective targeting of Bcl-2, alongside minimal effects on platelets, has led to the approval of venetoclax for the treatment of chronic lymphocytic leukemia [148]. In ER+ primary breast tumor xenografts venetoclax (ABT-199) showed similar activity to ABT-737, indicating the higher requirement for Bcl-2 rather than Bcl-xL downregulation for its activity [114]. These results pointed to the potential application of ABT-199 in ER+ breast cancer treatment, and in a phase I clinical trial comprising 33 ER+, Bcl-2-expressing metastatic breast cancer cases, venetoclax in combination with tamoxifen showed promising results. The overall response rate (ORR) was observed in 45% of patients, and the overall clinical benefit rate (CBR) reached 75%. Although the study was small, comparative analysis to studies including tamoxifen in first-line relapse showed favorable outcomes. Furthermore, combination therapy with venetoclax was well-tolerated with better toxicity profiles than other adjuvant anti-estrogen therapy such as mTOR, PIK3CA, and CDK4/6 inhibitors. Importantly, this study showed that in a subgroup of patients that previously received more than three lines of therapy for metastatic cancer, 67% showed clinical benefits from the combination with venetoclax, thus indicating Bcl-2 inhibition as a potential strategy to overcome tamoxifen resistance in previously unresponsive patients [136,149]. In a further phase II randomized clinical trial (Veronica) of venetoclax and fulvestrant, no benefits were observed in the inclusion of venetoclax in the treatment of metastatic breast cancer. This trial included 103 patients with ER+, HER2-, locally advanced/metastatic breast cancer following ≤2 lines of treatment (including a CDK4/6 inhibitor). The CBR of adjuvant venetoclax treatment did not exceed that of fulvestrant treatment alone [137,150].

Despite the upregulation of Bcl-2 upon anti-estrogen treatment, in long-term estrogen deprived cells (LTED), mimicking acquired resistance to aromatase inhibitor treatment, Bcl-2 levels were not induced. The study showed that short-term estrogen deprivation increased Bcl-2 levels. However, long-term deprivation decreased Bcl-2 levels. This suggested that short-term treatment with anti-estrogens through the increase of Bcl-2 sensitizes ER+ breast cancer cells to BH3 mimetics, whereas resistance acquisition leads to a decrease in Bcl-2 levels and shifts the resistance mechanism to other anti-apoptotic factors [151].

## 9. Targeting Pro-Survival Bcl-2 Family Proteins in Breast Cancer

### 9.1. Targeting Mcl-1 in ER+ Breast Cancer

**Figure 7:**
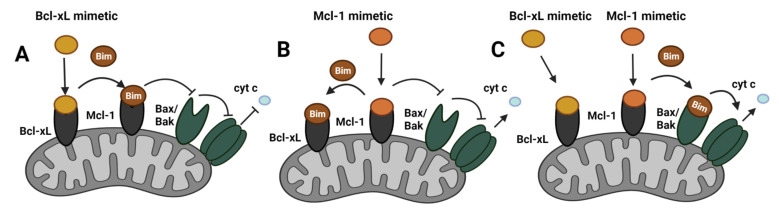
The compensatory role of Mcl-1 and Bcl-xL in resistance toward BH3 mimetics in ER+ breast cancer (**A**) The binding of the BH3 mimetic to Bcl-xL enables the translocation of Bim from Bcl-xL to Mcl-1, thus preventing the activation of Bak/Bax by Bim. (**B**) Binding of the Mcl-1 mimetic releases Bim, which binds to Bcl-xL. (**C**) The binding of both Bcl-xL and Mcl-1 with the corresponding BH3 mimetics enables the binding of Bim with pore-activating proteins Bax/Bak leading to their oligomerization and subsequent release of cytochrome c from the mitochondrial intermembrane space. Created with BioRender.com (accessed on 5 December 2021).

An important factor contributing to the resistance to Bcl-2/Bcl-xL mimetics has been associated with the upregulation of Mcl-1. In ER+ breast cancer cells, Mcl-1 levels were induced upon treatment with the Bcl-2/Bcl-xL inhibitor ABT-263, whereas *MCL1* silencing did not increase Bcl-2 or Bcl-xL, pointing to Mcl-1 as a driver of ABT-263 resistance. The upregulation of Mcl-1 in cells was shown to increase Mcl-1/Bim interactions, which were further induced upon ABT-263 treatment [123]. Anti-apoptotic Bcl-2 proteins have been reported to sequester activator BH3-only proteins, such as Bim, preventing the activation of Bak and/or Bax [152]. The inhibition of Bcl-2/Bcl-xL with the BH3 mimetic ABT-263 shifted the interactions of Bim to Mcl-1, suppressing its activation of Bax/Bak. Mcl-1 upregulation suppressed caspase3/7 activation, which indicated that ER+ breast cancer cells escape ABT-263-mediated cell death induction through Mcl-1 upregulation [123] (Figure 7A). One of the key drivers of Mcl-1 upregulation in breast cancer cells is the activation of PI3K/mTOR signaling. PI3K/mTOR signaling inhibition in *PIK3CA*-mutant and wild-type *PIK3CA* ER+ breast cancer cells reduced Mcl-1 levels. Downregulation of Mcl-1 upon mTOR inhibition was accompanied by a reduction in tumor volume, which was further reduced upon co-treatment with the Bcl-2/Bcl-xL inhibitor ABT-263 [138].

Further studies demonstrated the role of Mcl-1 in anti-estrogen resistance. Therapeutic strategies such as long-term estrogen deprivation increased Mcl-1 levels in cell lines and were confirmed in vivo. Mcl-1 upregulation rendered cells resistant to Bcl-2/Bcl-xL inhibition. The silencing of *MCL1* did not influence Bcl-2/Bcl-xL levels showing a lack of compensatory regulation of these proteins. However, *MCL1* silencing along with Bcl-2/Bcl-xL inhibition resulted in increased apoptosis induction in LTED cells in comparison to only Mcl-1 down-regulation. Fulvestrant treatment of LTED further upregulated Mcl-1, and the silencing of *MCl1* increased fulvestrant-mediated apoptosis in LTED cells, supporting Mcl-1 inhibition in anti-estrogen treatment [151]. The importance of targeting Mcl-1 was further demonstrated in studies showing its involvement in breast cancer-associated fibroblast-mediated resistance to the BH3 mimetics. Breast cancer-associated fibroblasts (bCAFs) comprise a large percentage of the breast stroma and can constitute up to 70% of the tumor volume. Research has shown that bCAFs promote the resistance of breast cancer cells to various therapeutic strategies, including chemotherapeutics such as doxorubicin and paclitaxel, used in the treatment of luminal B breast cancer [153]. Furthermore, bCAFs contribute to the development of hormone therapy resistance [154]. Recent research has shown that bCAFs suppress the pro-apoptotic effects of the BH3 mimetic ABT-737 and the BH3 mimetic ABT-199. These effects were not circumvented by the addition of anti-estrogens or chemotherapeutics. However, bCAFs increased Mcl-1 expression in breast cancer cells both at the mRNA and protein level, without affecting Bcl-2 levels. The down-regulation or activity inhibition of Mcl-1 desensitized cells to the effects of bCAF-mediated resistance to the BH3 mimetics. Thus the resistance of breast cancer cells to anti-apoptotic targeting of BH3 mimetics is associated with the influence of the stroma on the expression of Mcl-1. Moreover, the expression profiles of bCAFs showed that Mcl-1 and, to a lesser extent, Bcl-xL are responsible for the survival of bCAFS [155].

### 9.2. Compensatory Role Mcl-1 and Bcl-xL in ER+ Breast Cancer

The development of inhibitors targeting Mcl-1 is increasing in interest, and in recent years significant progress has been made in designing highly selective Mcl-1 inhibitors. Until now, 36 compounds have entered phase I clinical trials and are being evaluated for the treatment of recurrent or refractory hematologic malignancies [156,157]. In the case of breast cancer, Mcl-1 inhibitors have shown promising activity in preclinical studies. However, their role in the clinical setting is yet to be determined (Table 1). The selective Mcl-1 inhibitor VU661013 induced ER+ breast cancer cell apoptosis and inhibited tumor growth in vivo. VU661013 inhibited tumor growth by 25%, whereas no effects were observed with ABT-263. Combination treatment suppressed tumor growth to a greater extent than either agent alone, indicating the requirement of both Mcl-1 and Bcl-2/Bcl-xL inhibition for effective ER+ tumor suppression [138]. These findings were corroborated by another study using another specific Mcl-1 inhibitor, A-1210477, which increased the efficacy of ABT-263 in ER+ breast cancer cells. The synergistic activity between Mcl-1 and Bcl-2/Bcl-xL inhibition was shown through the disruption of Bim interactions with both Mcl-1 and Bcl-2/Bcl-xL, promoting apoptosis induction [125]. Furthermore, the analysis revealed that Bcl-xL is responsible for the observed synergistic effects of Mcl-1 and Bcl-2/Bcl-xL downregulation [125]. The overexpression of Bcl-xL, but not Bcl-2, reduced apoptosis induction in *MCL1*-silenced cells. Moreover, research suggests that Mcl-1 and Bcl-xL act redundantly in breast cancer cell survival, as the ratio of Mcl-1 to Bcl-xL was necessary for cell viability [125]. Further studies showed a synergistic induction in cell death through combined treatment of ER+ breast cancer cells with both an Mcl-1 (S63845) and a Bcl-xL (A-1331852) mimetic. Interestingly, sequential treatment with the Mcl-1 and Bcl-xL mimetics was important in eliciting an apoptotic effect. These studies further confirmed that Mcl-1 and Bcl-xL compensate for each other’s activity as inhibition of either protein leads to evasion of apoptosis. The inhibition of Mcl-1 with a BH3 mimetic shifts the binding of Bim to Bcl-xL, whereas blocking Bcl-xL results in the sequestering of Bim by Mcl-1 (Figure 7). These findings demonstrate the importance of targeting both proteins in ER+ breast cancer cells in order to avoid therapy resistance toward these BH3 mimetics [141].

### 9.3. Potential Drawbacks of BH3 Mimetics

Despite the promising preclinical value of Mcl-1 and Bcl-xL mimetics, research has suggested potential drawbacks associated with their clinical application. Mcl-1 is vital for the functioning of various cells, including those of the heart and brain [151]. Studies have shown that the deletion of *MCL1* influences the functioning of cells with an often detrimental outcome leading to cardiac failure [158,159]. Contrasting results were demonstrated with the inhibition of Mcl-1 activity. The use of a selective Mcl-1 inhibitor, S63845, displayed potent in vivo activity in a multiple myeloma xenograft model. No histomorphological changes were observed in organs at therapeutically efficacious doses. This discrepancy in the obtained results was explained by the fact that the physiological effects of intermittent pharmacological inhibition of Mcl-1 may not correspond to those exerted by irreversible *MCL1* knockout [139]. Bcl-xL, on the other hand, is essential in the functioning of hematopoietic and neuronal cells. The targeting of Bcl-xL with navitoclax was shown to result in side-effects such as thrombocytopenia, controlled by adjusting navitoclax dosing. Furthermore, since research has indicated the potential benefits of concomitant inhibition of Mcl-1 and Bcl-xL, this could have its consequences as these proteins not only play a redundant role in cancer survival but in such processes as neuronal development and hepatocyte survival [160]. Thus, their simultaneous inhibition may not be possible in the clinical setting. However, recent studies have demonstrated that concomitant pharmacological inhibition of Mcl-1 and Bcl-xL is feasible in vivo, utilizing an embryonic chicken model of rhabdomyosarcoma and zebrafish xenograft model of squamous cell carcinoma of the head and neck [161,162]. These findings encourage further research in determining the safety profile of targeting these Bcl-2 family proteins.

## 10. Role of Pro-Apoptotic Proteins in Breast Cancer-Mediated Cell Death

### 10.1. The Pro-Apoptotic Proteins Bax and Bak

The pore-forming proteins Bax and Bak play an essential role in mediating the activity of BH3 mimetics. Bak and Bax regulate the release of pro-apoptotic factors and are necessary for the execution of cell death. Bax/Bak-deficient tumors were shown to be resistant to Mcl-1 inhibition, whereas the presence of Bax/Bak in *MCL1*-deficient mice resulted in long-term tumor suppression [140]. Apoptosis induction through Mcl-1 down-regulation in breast cancer cells was reliant on the activity of Bak, not Bax. Bak has been reported to bind with Mcl-1 and Bcl-xL, but not Bcl-2 [163]. Mcl-1 inhibition resulted in Bak protein level increase, independent of *BAK* mRNA upregulation, and disrupted Mcl-1-Bak interactions. Furthermore, Bak silencing inhibited Mcl-1 downregulation-mediated apoptosis, indicating the requirement of Bak for eliciting apoptosis upon Mcl-1 activity inhibition [125].

### 10.2. The Pro-Apoptotic Protein Bik

Bcl-2 interacting killer (Bik) is a pro-apoptotic BH3-only protein. Bik is localized mainly to the outer endoplasmic reticulum membrane and participates in the intrinsic apoptotic pathway. Bik complexes with Bcl-2 and Bcl-xL, inhibiting their activity and inducing apoptosis in a Bax-dependent manner [164]. Bik participates in apoptosis induction mediated by anti-estrogens and estrogen starvation [95]. Low Bik levels rendered cells resistant to tamoxifen-induced cell death [165]. *BIK* silencing revealed its necessity for cell death induction in MCF-7/BUS cells (dependent on estrogen for growth). *BIK* mRNA and protein levels were induced in these cells upon estrogen deprivation or fulvestrant treatment. However, the levels of Bik target proteins Bcl-2, Bcl-xL, and Bax were not affected [95]. Similarly, tamoxifen induced Bik levels in MCF-7 cells [165]. Despite increased *BIK* mRNA expression in cells resistant to estrogen-induced apoptosis, Bik protein expression was not elevated [95]. In another study, upregulation of *BIK* mRNA upon fulvestrant treatment caused only a slight increase in apoptosis. The resistance to increased pro-apoptotic proteins was suggested to be associated with a concomitant increase in Mcl-1, which suppressed cell death [151].

Pandya et al. [166] reported Bik upregulation in breast cancer cells that induced moderate caspase activity and DNA damage without fully executed cell death, leading to failed apoptosis. Sublethal apoptosis led to heritable defective colony formation through the accumulation of mutations. Furthermore, long-term Bik expression generated cells with an aggressive phenotype, with increased migratory properties and stem-like properties. These findings correlated with clinical data from the analysis of 6 independent patient cohorts showing that high *BIK* mRNA and protein levels were a poor prognostic in ER+ breast cancer patients [166]. Studies have shown that failed or not fully executed apoptosis has oncogenic potential. In the process of failed apoptosis, low caspase activation occurs, inducing limited caspase-activated DNase (CAD) and caspase 3-dependent release of endonuclease G (EndoG), facilitating DNA double-strand breaks (DSB). This results in sustained genomic instability and oncogenesis [167], highlighting the effects of not fully executed apoptosis in cancer progression.

### 10.3. Tumor Suppressor p53

An important role in the upregulation of pro-apoptotic proteins is assigned to the activity of the tumor suppressor p53. P53 is induced upon cellular stress, facilitating either cell death or cell cycle arrest depending on the cellular context. P53 is regarded as the guardian of the genome as it prevents genomic instability by initiating cell cycle arrest allowing cells to perform DNA repair. Upon deleterious DNA damage, the activity of p53 is directed toward cell death through the induction of *BCL2* family gene expression as well as direct interactions with Bcl-2 family proteins [168,169]. In ER+ breast cancer cells, the activation of p53 was shown to be a requirement for Bax upregulation. P53 activation preceded the upregulation of Bax, which was alleviated in p53-null cells [170]. Furthermore, p53 was shown to activate Bax-mediated cell death in cooperation with BH3 mimetics. This mode of action was independent of the transcriptional activity of p53 and involved assisting the activity of BH3 mimetics by derepression of Bax from Bcl-xL and activation of released Bax. P53 was shown to enhance mitochondrial priming, increasing the activity of BH3 mimetics [171]. Another BH3-only protein regulated by p53 is Bik. The upregulation of Bik in ER+ MCF-7 breast cancer cells was associated with E2 deprivation or treatment with anti-estrogens [95]. This mechanism was connected with the activity of p53, as p53 was necessary for *BIK* induction at the mRNA and protein level by fulvestrant. This regulation of Bik by p53 did not involve the DNA-binding activity of p53. Interestingly, in another ER+ cell line, ZR75-1, Bik was shown to be constitutively expressed. However, its regulation was facilitated at the post-translational level through proteasomal degradation, highlighting the diverse mechanism of BH3-only protein regulation [172]. Further studies showed that ER+ breast cancers harboring wild-type p53 are resistant to p53-induced apoptosis in the presence of estrogen. ER antagonizes the pro-apoptotic activity of p53 by binding to a subset of p53 target genes involved in apoptosis induction. Apart from the ER agonist estrogen, partial antagonists such as tamoxifen inhibit p53-mediated gene expression, whereas full ER antagonists, such as fulvestrant, suppress the ability of ER to inhibit p53-mediated cell death. These findings suggest the involvement of certain anti-estrogens in the increased resistance of ER+ breast cancers to chemotherapeutic agents, which exert their activity through p53 induction. Since adjuvant treatment of early ER+ breast cancer frequently consists of chemotherapy followed by anti-estrogens, most commonly tamoxifen, and in menopausal women an aromatase inhibitor, this therapeutic sequence minimizes the effects of chemotherapy. Thus full ER antagonism (e.g., fulvestrant) and concomitant treatment with p53-activating agents are suggested in ER+/wild type p53 cancers to enable the pro-apoptotic activity of p53 [173].

## 11. Conclusions and Future Perspectives

Deregulation of Bcl-2 family protein expression/activity is a contributing factor in the sensitivity of ER+ breast cancer to treatment. Alterations in downstream estrogen signaling pathways mediated by anti-estrogen agents contribute to therapy resistance through the evasion of apoptosis. This is associated with the upregulation of pro-survival Bcl-2 family proteins, such as Bcl-2, Mcl-1, or Bcl-xL, or downregulation of pro-death Bcl-2 proteins. As demonstrated in the current review, Bcl-2 proteins are the point of convergence of various signaling pathways regulated by the estrogen receptors. The modulation of pathways regulating Bcl-2 family protein expression as well as the direct targeting of Bcl-2 proteins has offered an enhancement to the efficacy of currently clinically approved endocrine agents. Preclinical data regarding ER+ breast cancer treatment provides a strong rationale for the use of Bcl-2-targeted BH3 mimetics alongside endocrine therapy. However, a prerequisite in the efficacy of BH3 mimetics is the priming of cells for their activity. BH3 mimetics as single agents have been found ineffective in breast cancer treatment and require the upregulation of Bcl-2 complexes. This primed state of breast cancer cells with Bcl-2 complexes, which renders cells sensitive to BH3 mimetics, has been shown to be induced by anti-estrogen agents (e.g., tamoxifen and fulvestrant) as well as other ER+ treatment modalities such as PI3K/Akt inhibitors or chemotherapeutics. The concomitant use of BH3 mimetics with ER+ targeted therapy has been shown beneficial in preclinical studies, thus warranting their evaluation in the clinical setting.

The first Bcl-2 mimetics have entered clinical trials related to adjuvant ER+ breast cancer treatment. The first phase I clinical trial with the Bcl-2 inhibitor venetoclax and tamoxifen showed promising results. However, a phase II trial combining venetoclax with fulvestrant did not reveal the clinical benefits of adjuvant Bcl-2 inhibitor application. Due to the redundant role of Bcl-2 pro-survival proteins, preclinical research supports further investigation into targeting additional anti-apoptotic Bcl-2 family proteins. A major contributing factor to the resistance of Bcl-2-targeting mimetics, such as venetoclax, has been assigned to the compensatory upregulation of Mcl-1. The currently available clinically approved inhibitors that target signaling pathways regulating Mcl-1, such as PI3K/mTOR and MAPK inhibitors, have shown favorable outcomes in enhancing endocrine therapy. However, since various signaling pathways converge on Mcl-1, many possibilities of reestablishing its anti-apoptotic activity exist, thus enabling therapeutic resistance to treatment. This warrants further assessment of the clinical benefits of Mcl-1 inhibitors alongside other BH3 mimetics as the compensatory activity of anti-apoptotic Bcl-2 proteins, presented in this review, needs to be overcome for the full efficacy of BH3 mimetics. Further clinical investigations should shed light on the role of targeting Bcl-2 family members in ER+ breast cancer and address concerns regarding the safety of inhibiting these proteins. Moreover, the clinical relevance of Bcl-2 family protein expression levels in breast cancer patients could be an important inclusion in biomarker-guided therapy.

## Figures and Tables

**Table 1:** Effects of BH3 mimetics in ER+ breast cancer cells.

BH3 Mimetic	Target	Breast Cancer Model	Effects	Reference
ABT-263 (Navitoclax)	Bcl-2, Bcl-xL, Bcl-w	MCF-7 cell line	Synergistic activity with camptothecin, docetaxel, etoposide, rapamycin	Chen et al. [131]
ABT-737	Bcl-2, Bcl-xL, Bcl-w	ER+ PDX models, MCF-7 cells	Improved tumor and cell response to tamoxifen and PI3K/mTOR inhibitor	Vaillant et al. [114]
ABT-737	Bcl-2, Bcl-xL, Bcl-w	MCF-7 cell line	Sensitization of resistant cells to paclitaxel	Kutuk and Letai [134]
ABT-737	Bcl-2, Bcl-xL, Bcl-w	MCF-7 cells and ER+ primary breast tumor cells	Synergistic activity with the γ-secretase inhibitor GSIXII in apoptosis induction	Seveno et al. [135]
ABT-737	Bcl-2, Bcl-xL, Bcl-w	T47D	Increased activity of PI3K/mTOR inhibitor	Muranen et al. [133]
ABT-737	Bcl-2, Bcl-xL, Bcl-w	PDX model	Sensitization to docetaxel-mediated cell death	Oakes et al. [130]
ABT-199 (Venetoclax)	Bcl-2	ER+ PDX models, MCF-7 cells	Improved tumor and cell response to tamoxifen and PI3K/mTOR inhibitor	Vaillant et al. [114]
ABT-199 (Venetoclax)	Bcl-2	ER+, Bcl-2-expressing metastatic breast cancers	Phase I clinical trial of venetoclax in combination with tamoxifen; ORR 45% and CBR 75%	Lok et al. [136]
ABT-199 (Venetoclax)	Bcl-2	ER+, locally advanced/metastatic breast cancer	VERONICA: A randomized, phase II study of second-/third-line venetoclax + fulvestrant); No increase in CBR	Lindeman et al. [137]
VU661013	Mcl-1	MCF-7, T47D, HCC1428, MCF-7 xenografts	Increases apoptosis induction in cells and reduces tumor volume in combination with with ABT-263	Williams et al. [138]
A-1210477	Mcl-1	ER+ breast cancer cell lines	Synergistic antiproliferative activity with navitoclax	Xiao et al. [125]
S63845	Mcl-1	BT474 cell line	Increased cytotoxic activity of lapatinib	Kotschy et al. [139]
S63845	Mcl-1	*MMTV-PyMT* xenografts	Reduction of tumor growth	Campbell et al. [140]
S63845	Mcl-1	MDA-MB-415, T47D cell lines	Synergistic pro-apoptotic activity with Bcl-xL inhibitor	Alcon et al. [141]
A-1331852	Bcl-xL	MDA-MB-415, T47D cell lines	Synergistic pro-apoptotic activity with Mcl-1 inhibitor and AKT inhibitor (Ipatasertib)	Alcon et al. [141]

CBR, clinical benefit rate; ER, estrogen receptor; ORR, overall response rate; PDX, patient-derived xenograft.

## Data Availability

Not applicable.
